# Technological Tools and Artificial Intelligence in Estrus Detection of Sows—A Comprehensive Review

**DOI:** 10.3390/ani14030471

**Published:** 2024-01-31

**Authors:** Md Sharifuzzaman, Hong-Seok Mun, Keiven Mark B. Ampode, Eddiemar B. Lagua, Hae-Rang Park, Young-Hwa Kim, Md Kamrul Hasan, Chul-Ju Yang

**Affiliations:** 1 Animal Nutrition and Feed Science Laboratory, Department of Animal Science and Technology, Sunchon National University, Suncheon 57922, Republic of Korea; baushossain@gmail.com (M.S.); mhs88828@nate.com (H.-S.M.); keivenmarkampode@sksu.edu.ph (K.M.B.A.); eddiemarlagua@gmail.com (E.B.L.); gofkd20@naver.com (H.-R.P.); kamrul.ps@sau.ac.bd (M.K.H.); 2 Department of Animal Science and Veterinary Medicine, Bangabandhu Sheikh Mujibur Rahman Science and Technology University, Gopalganj 8100, Bangladesh; 3 Department of Multimedia Engineering, Sunchon National University, Suncheon 57922, Republic of Korea; 4 Department of Animal Science, College of Agriculture, Sultan Kudarat State University, Tacurong 9800, Philippines; 5 Interdisciplinary Program in IT-Bio Convergence System (BK21 Plus), Sunchon National University, Suncheon 57922, Republic of Korea; 6 Interdisciplinary Program in IT-Bio Convergence System (BK21 Plus), Chonnam National University, Gwangju 61186, Republic of Korea; yhkim8760@naver.com; 7 Department of Poultry Science, Sylhet Agricultural University, Sylhet 3100, Bangladesh

**Keywords:** estrus detection, technological tools, sow, posture recognition, smart farming

## Abstract

**Simple Summary:**

Accurate estrus detection by farmers is key to successful sow breeding as it offers the opportunity for timely mating, successful reproduction, and increased productivity. Traditional estrus detection methods based on physiological changes, mounting, and back pressure tests often fall short in accuracy, leading to lower conception rates and smaller litters. To address these issues, researchers are exploring modern technologies to study parameters such as vulvar temperature, posture, movement, and sound in relation to estrus. This review examines the effectiveness of these modern estrus detection techniques, and the findings indicate that they can enhance the accuracy of heat detection compared to conventional methods.

**Abstract:**

In animal farming, timely estrus detection and prediction of the best moment for insemination is crucial. Traditional sow estrus detection depends on the expertise of a farm attendant which can be inconsistent, time-consuming, and labor-intensive. Attempts and trials in developing and implementing technological tools to detect estrus have been explored by researchers. The objective of this review is to assess the automatic methods of estrus recognition in operation for sows and point out their strong and weak points to assist in developing new and improved detection systems. Real-time methods using body and vulvar temperature, posture recognition, and activity measurements show higher precision. Incorporating artificial intelligence with multiple estrus-related parameters is expected to enhance accuracy. Further development of new systems relies mostly upon the improved algorithm and accurate data provided. Future systems should be designed to minimize the misclassification rate, so better detection is achieved.

## 1. Introduction

With the increasing world population, animal product demand is also increasing [1]. Considerable efforts have been made over the past few decades, and global meat production has increased [2]. In that process, precision livestock farming (PLF) has gained tremendous attraction in the eyes of researchers and industries [3]. Laborious care of individual animals in large farms without the help of technology has made the PLF increasingly popular [4,5]. There are great provisions to introduce technological tools in the PLF system. Out of many branches of PLF, breeding pigs in healthy, depression-free conditions and noncontact forms has always been the target worldwide [6,7] as accurate and timely reproduction is essential to the swine business [8,9,10,11,12]. According to several studies, proper detection of estrus in sows plays a crucial role in predicting the correct insemination time and gaining desired production generation after generations [13,14,15]. Many researchers have emphasized timely insemination, stating “correct timing of insemination is very important for successful breeding farm” [16,17,18,19,20,21,22]. It is the sole opportunity within the 21-day estrous cycle when a female animal can mate, potentially leading to pregnancy and the initiation of reproductive activity [23]. An increase in the overall embryo implantation rate is evident from scientifically investigating sows’ estrus states [24]. Failing to recognize estrus at the right moment may hamper the conception rate and increase repeat breeders [16,25].

The average duration of a sow’s estrous cycle is 21 days, which may vary from 17 to 25 days depending on breed and individual differences [26]. The standing heat is variable, lasting from 24 to 96 h, and the average duration is 40 to 60 h [15,27]. Once two-thirds of estrus has passed, ovulation takes place [28,29]. To achieve maximum conception rate out of a cycle, it is recommended to perform insemination 0–24 h before ovulation [16,17,25,30,31]. If the process is not understood correctly, farmers may miss the best time for insemination, resulting in increased management and reproduction costs, lower conception and farrowing rates, as well as reduced litter size, compromising overall productivity [29,32,33,34,35,36,37,38,39,40,41]. Manual observation methods like the boar test method [42,43,44], standing estrus reflex checking [7,29,45], and looking for externally assessable physiological changes [46,47] are used conventionally by farmers to detect estrus. Out of the externally visible signs, release of vaginal mucosa and an enlarged, comparatively red-colored vulva are the main indicators of estrus in sows [28,48,49,50,51,52]. These signs are highly variable from individual to individual and cannot provide any information on the duration of standing heat. In addition, these processes are laborious and time-consuming [53]. Moreover, human observation cannot satisfy the requirements of real-time monitoring for 24 h [54]. To avoid economic loss on the investment in a single sow, she is required to produce at least three litters before culling [55]. However, many sows are replaced before they reach the break-even point [56]. In most cases, the removal of gilts is due to reproductive failure caused by a lack of observed estrus [57]. Increased replacement rate and decreased farrowing rate lead to increased non-productive days [58,59], and lower litters per sow per year (LSY) and piglets per sow per year (PSY) [60]. With a reduction in the non-productive day count from 35 to 24 days, a farm with 2400 sows can earn USD 528,000 additional revenue [61]. To address these problems, many researchers provided solutions through research on contactless and stress-free involuntary estrus detection in sows [62]. The use of accelerometers to monitor activity [46,63] and infrared thermal imaging for measurement of body and vulvar temperature [8,53,64,65,66,67,68,69] have been explored by researchers to identify estrus. Sensors connected with cameras are also being employed to monitor behavior and generate data for further processing by artificial intelligence to detect estrus and calculate the best possible insemination time for individual sows [70]. The current study is designed to compile the use of technological tools like accelerometers and other smart sensors, infrared thermography, smart cameras, and radio frequency identification supported by artificial intelligence in recognizing estrus. The objective of this review is to figure out the potentialities of those tools and models to facilitate further research. In the following section, the current use of technology in timely estrus detection is explained followed by the sections explaining the benefits and obstacles of using and popularizing them. Finally, this review sets the scope for future improvements.

## 2. Role of Technology and Artificial Intelligence in Estrus Detection

### 2.1. Sensors in Estrus Detection

**Figure 1:**
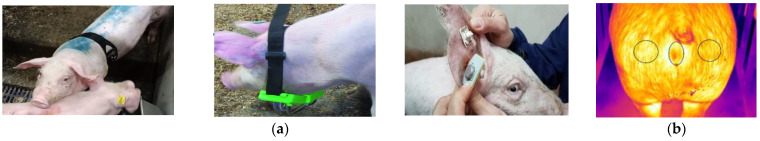
(**a**) Example use of sensors for data acquisition (**b**) points of infrared vulvar skin and gluteal skin temperature reading.

Ongoing scientific endeavors are in place to develop cost-effective, user-friendly methods for estrus detection [23]. Estrus detection can be more accurate in precision livestock farming as mechanical monitoring gives real-time data [71]. Using smart sensors set around animal pens and accelerometers suspended on sows’ necks or set elsewhere internally or externally has shown higher levels of physical activities on the day before estrus [62,72]. To see the difference in activity during estrus, a pedometer was used by Altmann [46], and he found that activity was twice during estrus than non-estrus days. Bressers [63] set the movement threshold to 10 m/s^2^ and found a 10-fold higher exercise of in-estrus sows than that of non-estrus sows using accelerometers. Geers et al. [73] also found the activity of sows to be 10 times higher before estrus. According to Roelofs et al. [74] a noticeable increase in activity is observed during estrus in animals. The daily mean activity of sows was examined using infrared sensors by Freson et al. [72] to recognize estrus. They concluded average body movement increased during estrus, with 86% accuracy and up to 10% false positive results. Johnson and Shade [11] used an internal accelerometer and an intra-vaginal data logger attached to an internal drug release device to investigate activity and found increased activity (37.8%, *p* < 0.01) on the day of estrus as compared to 3rd and 2nd day before estrus. Based on previous findings of increased activity during standing estrus a wireless accelerometer sensor was employed by Jeong and Yoe [75] to observe sows’ degree of activity. They also found increased activity of sows while in estrus. Some examples of sensor attachment are shown in Figure 1a.

Involuntary 24 × 7 monitoring of sows’ behavior aids in detecting heat and health conditions [76]. The invention of RFID (radio frequency identification) has made automated estrus detection possible for an individual sow within a group [77]. Ostersen et al. [7] monitored the time length and prevalence of sows’ visits to males using an RFID ear tag and a ticket window equipped with a sensor. The method detected 87.4% of the sows entering estrus with 99.4% specificity per sow day. Despite the very large specificity, the error rate was 91%. They concluded that accurately detecting estrus solely based on visits to the boar is challenging. Moreover, the spreading of African swine fever in recent years has influenced researchers to look for alternative ways rather than using males [78]. Søllested (2001) attempted to correlate sow visits to feeding stations with estrus. He found a non-significant correlation as eating behavior patterns of sows showed individual variability even in estrus [79]. The correlation between vaginal mucus electrical resistance and estrus onset has been examined by some studies [80,81]. The method initially gained interest, but the results differed significantly when various measuring locations in the vagina were explored and varied among individual sows as well [62,81,82,83,84,85]. Lee et al. [8] used an ultrasonic sensor array on the side of pens and DIRT (digital infrared thermography) on the back of pens to monitor sow postures and calculated the frequency of posture change. The overall standing period, standing number, and the number of sows standing for 10 min were increased (*p* < 0.05) by 25%, 32%, and 125%, respectively for the in-estrus period compared to the non-estrus period. They further continued the experiment and concluded a higher success rate of artificial insemination was observed when estrus was detected using their method over conventional methods.

Sensor-based systems reduce the need for constant human presence as they are designed to monitor 24 × 7 to detect early signs of estrus before visual cues become apparent. Using sensors integrated with Internet of Things (IoT) systems, farmers can remotely monitor estrus events. Despite the correlation found, factors like service length, durability, power consumption, individual differences, environmental interference, calibration challenges, and installment procedure negatively affected the popularization of these wearable tools in commercial swine farms and breeding industries. Variability in animal behavior and impaired sensitivity of sensors can contribute to inaccuracies. Improper placement and insecure attachment can also result in misinterpretations. Moreover, some behaviors may be subtle or challenging to detect accurately, leading to incomplete or inaccurate data.

### 2.2. Infrared Thermography in Estrus Detection

In the last few years, to detect the relationship between vulvar skin temperature (VST) changes and estrus initiation in sows, infrared thermography has been used [84]. Scolari et al. [53] and Simões et al. [68] used digital infrared thermography to detect temperature variability of vulvar skin and buttock skin of sows pre-estrus and during estrus; they found a significantly increased vulvar temperature at the initiation of estrus and significantly reduced vulvar temperature prior to ovulation. The two mostly observed sites for estrus detection via infrared thermography, and these are shown in Figure 1b. Since sows’ vulvas are exposed to the outside without being obstructed by their tail and touching is not required, fewer chances of disease spreading popularized infrared thermography [86]. Estradiol produced from developing follicles stimulates increased blood flow in the vulva through the internal pudendal artery, which contributes to raising the vulvar temperature [87,88,89]. Estradiol production peaks around 24 to 48 h before estrus in pigs [90]. Using digital infrared thermal imaging (DITI) Sykes et al. [69] observed that average and maximum temperatures of vulvar skin were significantly higher (*p* < 0.05) (33.4 ± 0.3 °C and 36.6 ± 0.2 °C, respectively) at the time of standing heat than those during diestrus (31.8 ± 0.6°C and 35.6 ± 0.3 °C, respectively). A similar figure was observed by Meng [91]. Chem et al. [87] measured the vulvar temperature of gilts in estrus and proestrus with DITI camera and concluded the average vulvar temperature was 34.5 °C during estrus and 33.7 °C in proestrus with a 0.8 °C margin of difference. Likewise, Weng [10] found a maximum temperature difference of 0.5 °C between the vulvar skin and the anterior udder skin on the day of estrus. Lee et al. [8] used DIRT and found that vulvar temperature increased by 20–32% during estrus. Jeong et al. [92] measured average rectal and vulvar skin temperature and found a non-significant difference (38.8 °C for in-estrus sows vs. 38.7 °C for non-estrus sows). Zheng et al. [9] used an infrared thermal imaging technique guided by YOLOV5s detector to monitor and detect sow estrus in real-time. The model detected estrus with 89.3% sensitivity, 94.5% specificity and 5.8% error rate. Johnson and Shade [11], using infrared thermography, found that the inner vaginal temperature in gilts decreased by 0.26 °C on estrus day as compared to 1, 2, and 3 days before estrus. Given that the method is non-invasive and round-the-clock observation is possible, many researchers have recommended it. There are portable infrared cameras with displays that make them easy to use for farmers compared to the processing of data generated by accelerometers or other motion-detecting sensors. However, infrared cameras are costly and less portable compared to other types of sensors. Processing infrared photos and extracting data can be complex requiring sophisticated image processing and interpretation methods. Both sensors and infrared thermography have been proven to be useful but there are still developments to make. Research on estrus detection based on sensors and infrared thermography in sows is presented in Table 1.

### 2.3. Artificial Intelligence and Machine Learning Models in Estrus Detection

Deep learning in recent years has gone through significant development and achieved success in the field of human action recognition [94,95,96]. Apart from human identification and tracking, information and communication technology (ICT) has been implemented in precision management and early detection of threats in animal farms as well [97]. The Internet of Things, photoelectric cells, 3D cameras, and sound recognizers backed up by artificial intelligence (AI) are gradually being introduced in smart farming [98,99,100,101,102,103]. These technologies in precision pig farming (PPF) improve farmers’ ability to operate large herds and strengthen the supervision of individual pigs’ status of fitness, welfare, and estrus [5,71,100,104,105]. Previously, vulva size and degree of vulva swelling were evaluated based on manual measurement or visual observation to detect estrus [106,107]. Depth cameras have proven their potential in measuring body parts, and body weight and in detecting behavior and condition [24,108,109]. Xu et al. [60] used machine vision technology to acquire a deep image of the vulva by light detection and ranging (LiDAR) camera to detect the changes in vulva size between proestrus and estrus. They concluded that vulva volume can be used as an authentic heat detection marker. Although the intensity and duration of vulva enlargement were highly variable among individuals, sows with naturally larger vulva showed the minimum response to an increase in size.

For precision pig farming smart equipment integrated with AI is presently applied in posture detection, activity measurement, and sound recognition [110,111,112]. With the progression of smart livestock industry and incorporation of AI, the usefulness and efficacy of photoelectric cells, infrared sensors and cameras have increased [113]. Recognition of sows behavior using image feature extraction, video analysis, and sow movement tracking has been explored by some researchers [114,115,116,117,118,119,120]. Zhuang et al. [121] analyzed pigs’ ear image data to look for the correlation between ear position and standing heat using a simplified Alexnet network structure. They reported that the duration of sows’ erected ears at the time of visiting males is a good index for standing heat detection. The sensitivity of the model applied was 79.16%. An ellipse fitting technique was applied by Nasirahmadi et al. [122] that resulted in 92.7% accuracy in identifying mounting events in sows. To solve the separation difficulty caused by occlusion, Li et al. [123] used Mask R-CNN (mask region-convolutional neural networks) to extract pigs individually and kernel extreme learning machine to detect the occurrence of mounting. The accuracy found was 94.92%. Yang et al. [124] developed an algorithm that resulted in 95.15% accuracy in mounting recognition using Faster R–CNN as pig locator and XGBoost as classifier. Li et al. [125] developed a convolution behavior recognition model (3D pig behavior recognition network) which achieved an accuracy of 97.63% in a test video dataset and 91.87% in a new test set collected from a completely different pigsty. Lei et al. [24] used a pseudo-male model that can produce the voice, scent, and touch of a real partner to identify estrus in post-weaning sows. They integrated machine vision to observe features like the contact duration of sows with bionic boars and the average duration of sows’ ears remained static. The average length of time for in-heat sows and the artificial males was found 29.7 s/3 min whereas diestrus sows’ contact time with bionic boar was only 8.44 s/3 min. Ears of in-estrus sows were static for an average of 41.3 s/3 min, which was higher than that of sows in diestrus. The experiment employed three distinct models, specifically the support vector machine (SVM), deep belief network (DBN), and sparse autoencoder (SAE). These models yielded recognition accuracy percentages of 90.00%, 96.12%, and 98.25%, correspondingly.

Sows go through dramatic hormonal changes before and during standing heat, which leads to behavioral expressions [126]. A decrease in sows’ rest time and an increase in recurrence and span of exercise are induced by hormonal flow. During estrus, peak estrogen levels elevate the level of a stress response hormone, namely cortisol [127,128,129,130]. Before the cortisol comes into action, the increased stress level increases heartbeats, breathing frequency, and blood pressure [131]. Consequently, the rising level of restlessness during estrus is understood to be the congenial response to increased estrogen levels [8]. A commercial product, Smart Sow Breeding (SSB), operated with an artificial intelligence backbone was implemented in three Belgian sow farms to observe the effectiveness of the system in detecting estrus timely and effectively. A total of 6717 reproductive cycles were studied to detect standing estrus automatically based on the recognition of behavioral changes of sows in estrus. The result exhibited that the implementation of the SSB system has a positive impact on reproduction success, in assessing heat signs, and helps in reducing required insemination count per estrus. Those field experiments found that estrus signs can be detected about 10–20 h earlier by the SSB system than by manual systems. They concluded by stating the use of SSB improves farrowing rate and litter size and decreases repeat breeding rate [27].

In recent times, the application of convolutional neural networks (CNN) in real-time object detection and classification has been implemented in many areas of surveillance and security [132]. Xue et al. [78] used the YOLO (you only look once) version 5s algorithm to develop a model for recognizing various postures such as standing, sitting, sternum laying, and lateral laying in sows. They also employed a convolutional block attention module to calculate the frequency of position changes. The model resulted in 94.1% accuracy in estrus detection with a precision level of 97.1%. They observed a significant increase in standing and sternum lying time during estrus. Daqin and Haiyan [133] used the Hopfield neural network estrus behavior identification model and concluded that the model can recognize estrus with 17% greater accuracy compared to manual methods, offering the additional advantages of timeliness and convenience.

Deep learning techniques have produced satisfactory results in sound identification in recent years [134,135]. Though most of the applications are made for human speech recognition, related models for animal voice recognition have also been developed [136,137]. Chen et al. [138] used a two-pronged AI model (VGG16-CNN and deep transfer learning and convolutional neural network) which satisfactorily (96.62% accuracy) distinguished between estrus calls and non-estrus calls. Wang et al. (2022) developed an estrus identification model relying on auditory input and deep CNN to detect estrus by accumulating and analyzing short and long recurrence of sounds. They found the model to be effective with 97.52% accuracy [139]. Increased accuracy, early detection, reduced labor requirement, and remote monitoring accessibility are the advantages offered by artificial intelligence. Most of the models discussed above have been operated under research conditions and were mostly limited to one single station. Large-scale implementation of models in different environmental conditions is needed to prove their true potential. While using AI, choosing parameter/s to be observed and machine learning model to be used are very important. Using multiple parameters can provide more reliable results than decisions based on a single parameter. Overall, the use of AI and machine learning (ML) showed better accuracy than sensors. Although data used by ML models are derived from different sensors; the predictive models and data processing capabilities of the developed algorithm make the difference. Research on estrus detection based on artificial intelligence and machine learning on sows is presented in Table 2.

## 3. Benefits of Technology and AI-Based Estrus Detection

Intensive labor, constant skilled observation, and manual handling like the back pressure test of sows are required in traditional estrus detection systems [53]. A large amount of money is spent as about 30% of total labor input is employed in detecting estrus [140]. To reduce this cost in large-scale farms automation is a must. Sow behavior and physiological parameters can be monitored in real-time using technological tools and AI-based models for timely and accurate estrus detection which directly affects breeding efficiency, conception rates, and litter sizes [29]. Moreover, reduced physical handling and non-invasive procedures reduce the stress on sows which ensures better animal welfare. Along with all these advantages, the number of non-productive days can also be minimized when estrus is detected timely [59]. Automated estrus detection using sensors, infrared thermography and machine learning models is not limited to sows only. Considerable progress is evident in cow [141,142,143,144], buffalo [23,145], and ewe [146,147] estrus detection as well. Though installing and implementing real-time estrus detection systems may require an initial investment, in the long run, will result in benefits and significant cost savings for livestock farmers and industries.

## 4. Challenges and Considerations

A significant upfront investment in equipment, software, and training is required to develop and implement technological tools in animal farms. Specially, small-scale farmers may find it difficult to invest the initial costs. Furthermore, adequate training is required to use and troubleshoot these systems. Tools like accelerometers are subjected to run out of battery and drop out from the animal body if not installed properly. Again, installing the accelerometers is a challenge in and of itself. Various environmental factors like ambient temperature, air movement, moisture, and debris can affect sensors and thermal images [69,87,148,149,150,151]. An ambient temperature of 20–30 °C is ideal for thermal imaging of animal skin [152,153]. At temperatures below 20 °C, fluctuations in vulvar skin temperature are difficult to identify [154]. Body hair covering thickness and hair color may also affect the accuracy of infrared thermography (IRT) readings [155,156]. Dirt or moisture presence in the measuring site can also affect IRT readings [93]. Even IRT can vary upon the time of the reading taken [157]. Developing accurate algorithms for automated estrus detection based on thermal patterns can be challenging.

For training machine learning (ML) models, large and high-quality datasets are required, which is challenging as they are mostly collected from external sensors, cameras, and microphones. Poor and biased training datasets and improper labeling can impact the overall performance of ML models. Due to individual variation of animals and farm to farm environmental variation, developing a universal model is challenging. Models require retraining to maintain accuracy. Installing new technology with existing farms can be challenging. Compatibility issues between different software and hardware components may arise. Performance of electronic equipment may be reduced in farms where the environment is harsh and dusty and where temperature fluctuations are frequent. However, with new and large commercial farms these limitations can be overcome as they can hire technical personnel for the operation of sophisticated models. The medium- and small-scale farmers need to be trained as well to ensure sustainable development in production.

## 5. Future Directions and Emerging Trends

In the coming days, miniaturization of sensors may lead to the invention of flexible and wearable sensors with higher sensitivity, wireless connectivity, and adaptivity to changing environments. Self-calibration will enhance the reliability and robustness of sensors where battery life will no longer be an issue as future sensors are supposed to be more power efficient and are even expected to harvest energy from the environment. Future sensors may simultaneously measure different parameters of interest. Connectivity and IoT devices will play a larger role in the real-time monitoring of sow conditions. The rise of edge computing will reduce the need for transmitting large amounts of data to centralized servers, improving response times and reducing network dependency. Blockchain technology may be employed to ensure the privacy of data, which can build trust between farmers and breeding industries.

More advanced and sophisticated algorithms for analyzing data from various sensors and sources are likely to be the features of future AI systems. Combining data from various sensors can provide a more accurate estrus detection profile. Integration of multiple sensing modalities, such as computer vision, Internet of things, sound recognition, and physiological monitoring will enable a more detailed assessment of sow behavior and health. Predictive models that forecast optimal breeding times based on historical data, environmental factors, and real-time monitoring will be developed using AI. The use of high-end devices with deep learning architectures like CNN and RNN (recurrent neural network) to minimize image processing time and maximize accuracy is expected. Easier interpretation of machine learning models for the farmers and practitioners will be offered through user-friendly interfaces like mobile applications. To tailor technology solutions to the specific needs of pig farmers, collaboration between researchers, technology developers, and the swine industry needs to be strengthened.

## 6. Conclusions

With the advances in technological sectors and increased demand for animal products, it is obvious that precision livestock farming is going to expand more in the future. The heart of PLF will be the use of synchronized technology. It is expected that large-scale commercial pig farms will adopt the technologies quite easily. However, wider adoption of technological tools and AI-based estrus detection systems in developing countries is required to improve food security and increase the efficiency of pig farming globally. Initially, farmers and farm attendants may struggle to cope with new technologies and AI systems. Ongoing support and training are necessary during the initial stages of implementation.

## Figures and Tables

**Table 1:** Sensors and infrared thermography in estrus detection of sows.

Technology/Model Used	Number of Observations	Breed	Parameters	Observations				Ref.
Pedometer	5 sows	-	Sow activity	Sows’ movement doubles during estrus				[46]
Accelerometers	-	-	Sow activity	Degree of movements and exercise increased 10 times				[63]
Intra-vaginal data loggers and internal accelerometer.	12 gilts	Yorkshire × Landrace	Vaginal temp. and activity	Temperature dropped by 0.26 °C (*p* < 0.01) at onset of heat Activity increased (*p* < 0.01; 37.8%) on estrus day				[11]
Accelerometer with WSN	Sows	-	Real-time sow activity	Sows in estrus increase activity 26–34 h after estrus initiation is right insemination time				[75]
Infrared sensor	58 sows	-	Daily activity	Peak daily activity has a greater correlation than average daily activity with estrus detection The accuracy found was 86% and 80%, respectively				[72]
RFID ear tag and sensor	39 sows	-	Time length and frequency of sow visiting male	Detected 87.4% of the sows entering estrus Specificity 99.4% False alarms 91.0%				[7]
Ultrasonic sensor array and DIRT camera	80 sows	Yorkshire × Landrace	Standing time, number, VST, and body temperature	Standing time and number increased (*p* < 0.05) VST in estrus increased by 20% (4 °C)				[8]
DIRT camera	25 gilts and 27 sows	Yorkshire × Landrace	VST and GST	VST and GST readings in sows were significantly higher (*p* < 0.05) than in gilts on a daily basis. 36 to 12 h prior to ovulation vulvar temperature fell significantly (1.5 °C; *p* < 0.05) in both sows and gilts.				[53]
DIRT camera	6 gilts and 30 sows	Large White × Landrace	Changes in VGT	Maximum VGT 5.3 ± 2.4 °C 48 h before Estrus Minimum VGT 0.5 ± 1.5 °C 12 h after estrus				[68]
DIRT camera	Sows	-	Body surface temperature	Thermal imaging cameras can be used for pig, cattle, and sheep estrus research				[93]
DIRT camera	32 gilts	Yorkshire × Landrace	VST	Condition	Max	Avg	Min	[69]
				Estrus	36.6 °C	33.4 °C	22.3 °C	
				Diestrus	35.6 °C	31.8 °C	20.7 °C	
				Difference	1.0 °C *	0.6 °C *	1.6 °C	
Infrared thermography	1000 sows	-	VST, GST, and eyes temperature	Vulvar temperature during estrus is higher				[91]
Infrared thermography	12 sows	Duroc	VST and rectal temperature	Higher vulvar and rectal temperatures during estrus Average temperature of two sites was 38.8 °C in estrus and 38.7 °C in non-estrus sows				[92]
DITI camera	7 gilts	(Landrace White × Yorkshire) × Duroc	VST	Condition	Max	Avg.	Min.	[87]
				Proestrus	35.8 °C	33.7 °C	26.5 °C	
				Estrus	36.9 °C	34.5 °C	29.4 °C	
				Difference	1.1 °C **	0.8 °C	2.9 °C **	
Infrared thermography	50 sows	Duroc	VST and UST	VST-UST reached the maximum (0.44 ± 0.068 °C) on sixth day post-weaning Delayed estrus detection results in smaller litter size (*p* = 0.005)				[10]
	50 sows	Landrace						
	32 sows	Large White						
DITI with improved FD-YOLOV5s detector	679 sows	Large White and Landrace	Vulvar temperature	Sensitivity 89.3% Specificity 94.5% Error rate 5.8%				[9]

WSN—wireless sensor network, RFID—radio frequency identification, DIRT—digital infrared thermography, VST—vulvar skin temperature, GST—gluteal skin temperature, VGT—vulvar temperature–gluteal temperature, *—*p* < 0.05, DITI—digital infrared thermal imaging, **—*p* < 0.001, UST—udder skin temperature (anterior upper part), FD-YOLOV5s—feature fusion and dilated convolution YOLOV5s; - indicates not given.

**Table 2:** Artificial intelligence and machine learning models in estrus detection for sows.

Technology/Model Used	Number of Observations	Breed	Parameters	Observations			Ref.
LiDAR camera with customized algorithm	7 sows	Landrace	Vulva size	Change in vulva size was relatively less in sows with naturally larger vulvas Individual variation is high			[60]
	1 gilt	Landrace × Yorkshire					
Alexnet neural network	Sows	Large white	Duration of ear erection	Sensitivity 79.16%			[121]
Machine vision models (DBN, SAE and SVM) and bionic boars	76 sows	Yorkshire	Contact time to bionic boar and ear position	Accuracy: DBN-96.12%, SAE-98.25%, SVM-90.00%			[24]
				Condition	Contact duration	Static ear duration	
				Proestrus	8.44 s/3 min	-	
				Estrus	29.7 s/3 min	41.3 s/3 min	
SmaRt Sow Breeding (PigWatch)	750 sows	TN70	Behavioral data	Estrus detection 10–20 h earlier than the farmer Improve FR%, FRFI% and NTBP and reduce RB% Provide less variable results in estrus duration The number of inseminations per estrus decrease			[27]
	250 sows	Landrace × large White					
YOLOV5s model and CBAM	72 sows	Yorkshire × Landrace	Frequency of posture change	Increase in standing and sternum laying and decrease in lateral laying time while in estrus Precision 97.1%, accuracy 94.1%, image processing time 74.4 ms			[78]
MobileNetV3_esnet model with deep CNN algorithms and Log-mel spectrograms	63 sows	Canadian	Short and long frequency estrus sound	The model acquired 97.12% precision, 97.34% recall, 97.59% F1-score and 97.52% accuracy			[139]

LiDAR—light detection and ranging, DBN—deep belief network, SAE—sparse autoencoder, SVM—support vector machine, FR—farrowing rate, FRFI—farrowing rate after first insemination, NTBP—number of total born piglets per litter, RB—repeat-breeders, YOLOV5s—you only look once version 5s, CBAM—convolutional block attention module, CNN—convolutional neural network; - indicates not given.

## Data Availability

No new data were created or analyzed in this study. Data sharing is not applicable to this article.
